# Experimental Animal Models of Phenylketonuria: Pros and Cons

**DOI:** 10.3390/ijms26115262

**Published:** 2025-05-30

**Authors:** N. A. Bobrova, D. I. Lyubimova, D. M. Mishina, V. S. Lobanova, S. I. Valieva, O. N. Mityaeva, S. G. Feoktistova, P. Yu. Volchkov

**Affiliations:** 1 Federal Research Center for Innovator and Emerging Biomedical and Pharmaceutical Technologies, 125315 Moscow, Russia; bobrova_na@academpharm.ru (N.A.B.); feoktistova_sg@academpharm.ru (S.G.F.); volchkov_py@academpharm.ru (P.Y.V.); 2 Moscow Center for Advanced Studies, Kulakova Str. 20, 123592 Moscow, Russia; lyubimova.di@genlab.llc (D.I.L.); mishina.dm@genlab.llc (D.M.M.); 3 Morozov’s Moscow City Children Clinical Hospital, Beregovoy Proezd 5A/1, 121087 Moscow, Russia; kakaulinavs@gmail.com (V.S.L.); svalieva@morozdgkb.ru (S.I.V.); 4 Faculty of Fundamental Medicine, Moscow State University, Lomonosov Sky Pr., 27, 119991 Moscow, Russia; 5 Moscow Clinical Scientific Center N.A. A.S. Loginov, 111123 Moscow, Russia

**Keywords:** phenylketonuria, metabolic disorder, phenylalanine, phenylalanine hydroxylase, animal models

## Abstract

Phenylketonuria (PKU) is a common inherited metabolic disorder characterised by impaired metabolism of the amino acid phenylalanine. The disease results from a mutation in the phenylalanine hydroxylase (PAH) enzyme, which converts phenylalanine (Phe) into tyrosine (Tyr). The absence or inactivity of this enzyme results in significantly elevated levels of Phe in the blood, which can lead to severe neurological conditions, including intellectual disability, epilepsy, and other developmental disorders. Since its discovery, animal models have played a crucial role for understanding the pathophysiology of PKU, as well as providing recognisable proof of targets and surveying new remedial specialists and in vivo medicines. In the present study, we conducted a comprehensive review of the experimental and non-experimental animal models employed for phenylketonuria and its associated complications.

## 1. Introduction

Phenylketonuria (PKU) is one of the most prevalent inherited metabolic disorders, characterised by aberrant metabolism of the amino acid phenylalanine. This condition arises from a deficiency of the enzyme phenylalanine hydroxylase (PAH), which is essential for the conversion of phenylalanine (Phe) to tyrosine (Tyr). Absence or decreased activity of this enzyme results in significantly elevated blood levels of Phe, which can lead to serious neurological conditions including intellectual disability, epilepsy, and other developmental disorders [1,2].

Following the discovery of the condition in the mid-twentieth century [3,4], scientific research has focused on the development of effective diagnostic and therapeutic methods. This has included the creation of various experimental animal models, which have facilitated a deeper comprehension of the pathogenesis of PKU and the assessment of potential therapeutic strategies. These models facilitate comprehensive analyses of the molecular and biochemical mechanisms underlying the disease, as well as the evaluation of the efficacy of new pharmacological drugs and therapies. The development of these models typically involves genetic modification or reduction of the enzyme activity, thereby simulating phenylketonuria in humans. A variety of animal species, for example, mice and pigs [5,6], are used to create these models. Each of these animals has its own advantages and disadvantages, which must be taken into account when selecting the optimal model for specific studies.

The study of phenylketonuria and other inherited metabolic disorders requires an understanding of the genetic, biochemical, and molecular aspects involved. Experimental models are central to this process, allowing researchers to carry out experiments that would otherwise be unethical to perform in humans. This approach contributes to the understanding of disease mechanisms and to the evaluation of new therapeutic approaches, including pharmacological correction of metabolic disorders and gene therapy.

Nevertheless, the utilisation of experimental models is not without its challenges. Primarily, it is imperative to recognise that disparate metabolic pathways may be exhibited by distinct animal species, thereby rendering the extrapolation of findings from one model to another a challenging endeavour [7].

It is therefore important to consider the role of experimental animal models of phenylketonuria in advancing our understanding of the disease and developing effective therapies. The present study is an extensive critical review of existing PKU animal models, assessing their strengths and limitations. By systematically comparing traditional rodent models with alternative species, such as zebrafish and avian models, this study addresses knowledge gaps concerning the suitability of different models for various research objectives. This study provides researchers with a valuable resource for refining PKU models and developing more effective, ethically responsible therapeutic strategies.

## 2. Phenylketonuria

PKU was first described in 1934 by the Norwegian physician Ivar Asbjørn Følling, who observed a high concentration of phenylpyruvic acid in the urine of patients with intellectual disability [4,8]. He established a correlation between this biochemical finding and the patients’ cognitive impairments, proposing that the disorder may have a metabolic origin. Følling’s research into the characteristic musty odour in the urine of affected individuals, caused by the accumulation of Phe metabolites, was crucial for the initial recognition of PKU as a distinct clinical entity. However, at the time, the disease was known as ‘imbecillitas phenylpyruvica’, and it was not until several years later that English geneticist Dr Lionel Penrose renamed this metabolic disorder phenylketonuria because of the characteristic green colour of phenylpyruvic acid [4].

Despite Følling’s discovery, PKU remained relatively obscure until the 1960s, mainly due to the lack of reliable diagnostic methods for infants and children. The identification of Phe in urine and its association with intellectual disability was a major breakthrough. However, the technology to systematically assess Phe levels in blood or urine did not become available until much later [9]. In 1961, Dr Robert Guthrie developed a method of blood spot analysis and gave a presentation on phenylketonuria to the local chapter of the Association for Children with Developmental Delays (Jamestown, New York) [10]. This was the beginning of newborn bloodspot screening (NBS). Since then, PKU is typically diagnosed through NBS programmes. Early detection and dietary intervention are of paramount importance to prevent the neurological complications associated with the disorder.

The clinical manifestations of PKU vary according to blood Phe concentrations, which are dependent on PAH enzyme activity. There are three categories based on Phe concentrations: classic PKU (above 1200 μmol/L of Phe), the most severe form, is characterised by almost no PAH activity and thus requires strict dietary management. This form of PKU, the most severe form, is predominant in Eastern Europe, Turkey, and Australia (over 70% of cases). The other two categories are mild PKU (600–1200 μmol/L) and mild hyperphenylalaninemia (HPA, 120–600 μmol/L). The prevalence of these two forms is higher in Western Europe, North America, and parts of East Asia, and may permit greater dietary flexibility due to the presence of residual enzyme function [2,11].

### 2.1. Epidemiology of PKU

Globally, PKU is among the more common inherited disorders, occurring in approximately 1 in 23,930 newborns [11]. However, the prevalence of the condition varies significantly around the world. For example, in Europe, PKU is among the most common inherited metabolic disorders. Prevalence rates range from approximately 1 in 2700 to 1 in 4500 newborns in countries such as Ireland and Italy, to less than 1 in 100,000 newborns in Finland [12]. The United States reports an incidence of 1 in 13,500 to 1 in 25,000 newborns. In Asia, PKU prevalence is significantly lower, with Japan and Thailand showing some of the lowest rates worldwide (1 in 125,000 and 1 in 227,273, respectively). China, however, has a relatively higher prevalence of 1 in 15,924 [11,13].

### 2.2. Genetics and Pathophysiology

PKU is an autosomal recessive condition, which means that for a child to manifest the condition, both parents must carry and pass on a defective gene. PAH is encoded by the *PAH* gene, which is located on chromosome 12q23.2, and its function depends on the presence of cofactors and proper structural organisation [2]. PAH is a homotetrameric enzyme, meaning it consists of four identical subunits. Each 52 kDa subunit has three primary domains: regulatory, catalytic, and oligomerisation domains. The first found at the N-terminal (RD, residues 1–110) with an unstructured N-terminal tail (N term; residues 1–29), this domain modulates enzyme activity and responds to Phe concentrations in the blood. The second is the central region (CD, residues 111–410) of the enzyme, which contains the active site where Phe hydroxylation occurs. The last, located at the C-terminal (OD, residues 411–453), facilitates the assembly of the tetramer, which is necessary for optimal enzymatic activity [12].

**Figure 1:**
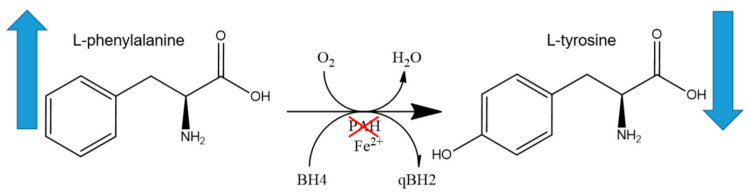
Metabolic pathway of PKU: Phenylalanine hydroxylase (PAH) catalyses the hydroxylation of L-phenylalanine to L-tyrosine. In the case of phenylketonuria, the reaction fails, and phenylalanine levels increase (the arrow up) and tyrosine levels fall (the arrow down).

The catalytic activity of PAH is contingent on three factors (Figure 1). Firstly, the presence of tetrahydrobiopterin (BH4) is essential as a cofactor, providing the reducing equivalents necessary for the hydroxylation reaction. Secondly, molecular oxygen is indispensable for the introduction of a hydroxyl group to Phe. Thirdly, non-heme iron, located in the active site, facilitates electron transfer during catalysis [1,12].

Elevated blood Phe has been demonstrated to possess neurotoxic properties, but the precise mechanisms underlying neurotoxicity remain unclear. It is hypothesised that elevated blood Phe causes defective synapse formation, impairment of neuronal cell growth, and abnormal myelination, the latter of which persists throughout life [14]. Dietary Phe restriction may reduce the risk of neurotoxicity if initiated within the first few weeks of life. A robust correlation between control of blood Phe in childhood and intelligence quotient (IQ) has been demonstrated [14].

Tyrosine plays a key role in neurotransmitter synthesis, including the production of dopamine, epinephrine, and norepinephrine. Reduced levels of tyrosine have been associated with attention deficit hyperactivity disorder (ADHD) [3,14]. In addition to its role in neurotransmitter synthesis, tyrosine has a number of other metabolic functions. These include conversion to thyroxine in the thyroid gland, conversion to melanin in melanocytes, and complete catabolism to acetoacetate (a ketone) and fumarate (a Krebs cycle intermediate), which are then used as fuel [1].

PAH deficiency in PKU manifests primarily as neurological symptoms due to elevated levels of Phe in the blood, which crosses the blood–brain barrier and accumulates in the brain. In the absence of intervention, elevated Phe levels can result in severe neurological impairments, including intellectual disability, microcephaly, seizures, and behavioural disturbances [14]. These effects are attributable to the neurotoxic buildup of Phe, which interferes with normal brain development and function. The only notable non-neurological indicator of PAH deficiency is hypopigmentation, which arises from impaired melanin synthesis due to diminished tyrosine availability [1].

Elevated Phe levels have been shown to competitively inhibit the transport of large neutral amino acids, such as tyrosine and tryptophan, into the brain. This disruption has been shown to result in a reduction of the synthesis of key neurotransmitters, such as dopamine and serotonin, which are critical for maintaining normal cognitive and emotional function [15]. Consequently, untreated PKU can result in long-term deficits in cognition, behaviour, and neurological stability.

Some research has demonstrated that individuals with phenylketonuria (PKU) manifest structural brain abnormalities, particularly in the cerebral white matter [1,14,16]. This finding has been consistently replicated in neuroimaging studies. These include demyelination and reduced white matter volume, likely due to Phe-induced toxicity affecting oligodendrocyte function and myelin formation. In addition, recent research suggests that phenylalanine may self-assemble into amyloid-like fibrils, which resemble those found in neurodegenerative conditions such as Alzheimer’s diseases [17]. This process may contribute to neuronal damage and cell death in PKU.

Even with early dietary management, individuals with PKU often face persistent cognitive and neurological difficulties. These include impairments in executive function, attention, processing speed, and working memory [18]. Elevated Phe continues to affect cerebral myelin, protein synthesis, and neurotransmitter levels, contributing to these cognitive challenges [1]. Advanced neuroimaging has revealed that early-treated PKU patients frequently display reduced overall brain volume, particularly in the parietal and occipital cortices, and increased volume in the putamen—alterations that correlate with cognitive deficits [19]. Furthermore, even short-term elevations in Phe can cause transient reductions in grey matter and compromise white matter integrity, highlighting the necessity of maintaining strict metabolic control throughout life [20].

## 3. Animal Models

Animal models are essential in phenylketonuria (PKU) research, providing critical insights into the pathophysiology of the disease and facilitating the development of therapeutic strategies. By using animal models, researchers can mimic the human condition, allowing them to investigate disease mechanisms and evaluate potential treatments in a controlled environment.

Several animal models have been developed to study PKU, each offering unique advantages. Mouse models have been genetically engineered to lack PAH activity, closely mimicking the human disease phenotype. These models have been instrumental in understanding the neurological deficits associated with PKU and in testing dietary and pharmacological interventions. However, traditional rodent models have limitations, as they may not display the full spectrum of PKU symptoms observed in humans. More recently, zebrafish (*Danio rerio*) have emerged as a complementary model due to their genetic homology to humans, rapid development, and suitability for high-throughput drug screening. While these models provide valuable insights, species-specific metabolic differences must be considered when translating findings to human clinical applications. To address this, researchers have developed alternative models, such as the pig, which has closer physiological similarities to humans. This model exhibits elevated blood Phe levels and neurological abnormalities, providing a promising platform for preclinical studies.

### 3.1. Mouse Models

Mice (*Mus musculus*) have played a pivotal role in biomedical research for over a century, serving as a fundamental model for understanding human biology and disease mechanisms. The genetic, anatomical, and physiological similarities between mice and humans render them an invaluable model for studying a wide range of human conditions [21].

Currently, there are numerous mice models (Table 1), which have been created using different methods, such as chemically induced mutation, or CRISPR/Cas9.

During the 1970s and 1980s, both mouse and rat models were utilised in the study of phenylketonuria. These models were based on increasing Phe levels in the body by adding the amino acid to the diet or by systemic administration of enzyme inhibitors (parachlor-Phe; alpha-methyl-Phe) or combinations of these methods.

The initial models had several significant limitations. For example, the addition of Phe to the diet led to an increase in tyrosine concentration rather than a decrease, which is characteristic of phenylketonuria.

In 1993, Shedlowski and his team [6] created the most well-known and widely used Enu2 model, utilising the mutagen N-ethyl-N-nitrosourea (ENU). The advent of this mouse model has been instrumental in fostering a more profound comprehension of PKU at both the biochemical and behavioural levels. It has also facilitated the evaluation of innovative therapeutic interventions, such as enzyme substitution and genome-based editing. Subsequently, novel mouse models began to be developed.

#### 3.1.1. BTBR-Pah^enu2^/J

This mutation is chemically induced using the mutagen N-ethyl-N-nitrosourea (ENU). It results in the substitution of one amino acid, F263S, which causes a dramatic decrease in the catalytic activity of PAH in homozygotes and is a model for a severe form of phenylketonuria.

In contrast to Enu1, this mutation has a much more severe form of hyperphenylalaninemia, in which the content of Phe in serum is increased 10–20 times, as well as in adult individuals increased concentration of ketones in the urine. Homozygotes demonstrate a marked reduction in growth rate and head size. Behavioural abnormalities emerge from two weeks onwards. These behavioural abnormalities include, but are not limited to, frequent rolling over during grooming, impaired coordination during swimming tests, and a general decrease in alertness.

It is noteworthy that mice exhibiting this mutation manifest hypopigmentation, which becomes noticeable at two weeks and intensifies by weeks five to six. Hypotrichosis manifests itself at an older age if the diet is not low in Phe.

Homozygous females retain their fertility, but with a reduced number of pups in the litter, these pups are prone to perinatal death [6].

#### 3.1.2. B6.BTBR-Pah^enu2^/MalnJ

In the absence of dietary maintenance, homozygotes of this model manifest a severe form of phenylketonuria, characterised by the development of seizures and other neurological symptoms, and are characterised by hypopigmentation. Homozygotes are viable, but their quality of life is significantly impaired, with females being unable to bear offspring.

Furthermore, Jackson Laboratory has reported instances of ocular abnormalities, including corneal opacity and enlarged eyes. Histological analysis has revealed cases of lens rupture, anterior uveitis, and glaucoma [28].

#### 3.1.3. BTBR-Pah^enu1^/J

The chemical induction of a mutation, similar to that observed in ENU2, results in a milder form of hyperphenylalaninemia. This conserved V106A point substitution in the N-terminal region of PAH results in a milder phenotype than the Pah^enu2^ mutation. Homozygotes manifest delayed excretion of Phe when administered intravenously [23].

In contrast to Pah^enu2^, this model manifests a less severe form of hyperphenylalaninemia. Homozygotes manifest no hypopigmentation or neurological abnormalities. Furthermore, homozygous females of this model demonstrate increased fertility, a trait that is not observed in Enu2. It is also noteworthy that the level of Phe in blood serum is close to normal and exceeds normal levels by only twice in the brain [23].

#### 3.1.4. B6(Cg)-Pah^tm1.1(PAH*R408W)Xiwan^/J

Humanised R408W mice have been found to be carriers of human *PAH* exon 12 with the R408W mutation, which is the conversion of arginine to tryptophan, embedded in the *PAH* gene. The homologous R408W mutation in humans is associated with PKU. This mutation has been identified as the most prevalent in the human population.

In this model, blood Phe levels average more than 1200 µmol/L, leading to the development of neurological symptoms including seizure syndrome [24].

Mice that are homozygous for this mutation are viable and fertile, but the number of pups in the litter is reduced, and the offspring die either intrauterine or within the first seven days after birth. Furthermore, these mice are characterised by a reduction in size relative to the standard mouse. Hypopigmentation is observed due to decreased melanin synthesis. Furthermore, the levels of Phe in homozygotes range from 1000 to 1500 µmol/L. This model can be used in testing humanised therapeutics [24].

#### 3.1.5. Mouse Models C57BL/6J-Pah^em1Xiwan^/J (PAH P281L)

This model constitutes mice that carry the P281L mutation (wherein proline is converted to leucine) in the *PAH* gene [25].

The CRISPR/Cas9 endonuclease-mediated homologous repair of *PAH* was utilised to introduce an amino acid substitution at position 281, thereby converting proline to leucine (P281L, c.842 C-T). Mice homozygous for this congenital mutation are viable and fertile, but homozygous females have fewer offspring, and the pups usually die in the perinatal period. Homozygotes exhibit elevated blood Phe levels, hypopigmentation due to decreased melanin synthesis, and are smaller in size. The blood Phe levels of these subjects range from 1455 to 2242 µmol/L, in comparison to the 120 µmol/L levels observed in the control group, which is analogous to human values [25].

#### 3.1.6. PAH-KO

This model was developed from the C57BL/6j line using CRISPR/Cas9, where codon 7 (GAG) in the *PAH* gene was replaced with TAG, resulting in the conversion of GAG (Glu) to TAG. The objective of this modification was to create a model that would not produce the endogenous PAH protein [26].

During the process of growth, the weight of homozygotes was found to be lower than that of heterozygotes of this model. Furthermore, a significant elevation in Phe levels was observed, accompanied by a reduction in tyrosine levels.

After euthanasia at 6 months of age, the phenylalanine protein was not detected by Western blot analysis, and the absence of this protein was confirmed by immunohistochemistry. The brains of homozygous mice contained higher levels of phenylalanine and reduced levels of neurotransmitters and tyrosine, which is characteristic of humans with phenylketonuria. As with other models, PAH-KOs show reduced body weight and brain weight, and behavioural abnormalities [26].

#### 3.1.7. Pah-R261Q

The mouse model was created by utilising CRISPR/Cas9 genome editing technology to introduce the p.R261Q-PAH mutation. This mutation has been shown to result in the manifestation of hyperphenylalaninemia in mice. Phenylalanine levels have been found to be elevated to twice the normal levels. It is noteworthy that the patient does not exhibit other phenotypic abnormalities that are characteristic of classical phenylketonuria, such as hypopigmentation. Furthermore, Pah-R261Q males exhibited higher body weight in comparison to the wild type, and all mutant mice demonstrated a reduced resting respiratory exchange ratio (RER), suggesting a metabolic shift towards fat and protein oxidation. A comprehensive metabolic analysis of Pah-R261Q mice was conducted, which revealed mild hyperphelaninemia, accompanied by a concomitant decrease in L-Trp, L-Tyr, and their metabolites. The analysis also revealed dyslipidaemia, signs of insulin resistance, and oxidative stress. Pah-R261Q analysis revealed the preservation of neurological function in severe hepatic BH4 deficiency. Excessive and prolonged elevations in L-Phe levels were observed in mutant mice following periods of physical exertion, reaching levels approximately 10-fold higher than baseline. These levels correspond to moderate HFA in humans. In contrast, control groups comprising heterozygotes and wild-type (WT) mice exhibited a significantly weaker response. The efficacy of BH4 therapy: A 4-day course of BH4 was found to result in a statistically significant reduction in hyperphenylalaninemia. A 28% reduction in AUC was observed, thus confirming the BH4-sensitive phenotype. The results obtained correlate with clinical data in patients with the R261Q mutation. Despite the presence of similar residual PAH activity (~15% of normal), humans with the R261Q mutation exhibit more severe hyperphenylalaninemia (HPA) (L-Phe > 600 μM) than mice (~110 μM). This discrepancy can be attributed to differences in the regulation of PAH expression in different species [27].

Mouse models are a crucial research tool due to their small size, high reproductive rate, and ease of genetic manipulation, making them the most commonly used mammals for studying human diseases. Although evolutionary differences between mice and humans—such as disparities in size, metabolic rate, lifespan, and immune system—exist, their overall genetic and physiological similarities make them valuable in biomedical research. In the context of phenylketonuria (PKU), mouse models have been instrumental in uncovering disease mechanisms and testing therapeutic approaches. However, despite their contributions, there is increasing interest in developing alternative models that more accurately replicate human PKU pathology. Genetically humanised mouse models, which incorporate human gene sequences, are being explored to provide more precise platforms for evaluating gene-editing techniques and other targeted interventions. Nonetheless, limitations such as differences in phenylalanine metabolism, mild cognitive impairment compared to human PKU patients, and challenges in studying long-term disease effects underscore the need for complementary models.

### 3.2. Pig Models

**Figure 2:**
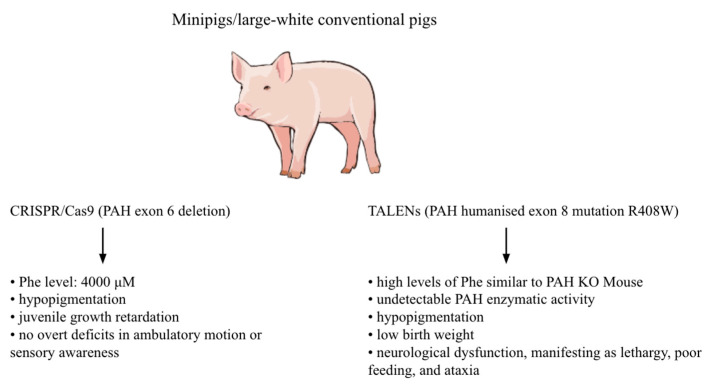
Pig models for the study of phenylketonuria.

PAH-deficient pig models enable assessment of new therapeutics in a large-animal model during the preclinical drug development stage, providing a greater measure of safety before human trials. It allows studies to comparatively estimate the neuroanatomical and functional correlates of PKU and whether differences in treatment lead to neurologically distinct outcomes. In other cases, pigs are utilised to facilitate the development of new therapeutic candidates, including gene-editing approaches, and to enable preclinical testing of low-phenylalanine diets. Thus, current research confirms the success of using pigs as models (Figure 2) [5,29,30].

A PAH-deficient PKU model was created using the CRISPR/Cas9 system by in vivo zygotic genome editing. Hybrid porcine zygotes of domestic and Yucatan minipig background were microinjected with Cas9 mRNA and two sgRNAs in two experiments. Following 5 days of in vitro growth, 57 blastocysts were genotyped for the presence of genome-editing deletions, and 19 of them were used for embryo transfer to a surrogate sow. Not all blastocysts yielded sufficient DNA for amplification of a PCR product, but the data imply a minimum frequency of approximately 48% blastocysts with a detectable *PAH* exon 6 deletion and indicate a high degree of in vivo genome editing. Two embryo transfer attempts were then made using separate batches of microinjected embryos, from which a single founding litter of PKU swine was successfully generated [5].

The piglets were monitored throughout their first year of life. Biochemically, they possessed a classical PKU phenotype, with extraordinarily high blood and urine Phe concentrations, along with secondary urinary markers of HPA. Intriguingly, Phe homeostasis in the affected pig was consistently greater than 4000 μM, which is greater than the Pah^enu2^ mouse and well in excess of untreated patients with classical PKU. Phenotypically, the affected pig had hypopigmentation and juvenile growth retardation but did not exhibit the devastating neurocognitive and neurological clinical characteristics of untreated PKU, despite apparent brain MRI abnormalities. The pig did not have any overt deficits in ambulatory motion or sensory awareness [5].

Another model was designed using TALENs, targeting exon 8 of the *PAH* allele in porcine fibroblasts, combined with a single-stranded oligodeoxynucleotide (ssODN) template. The humanised R408W mutation and human SNPs were introduced. Early-passage embryonic fibroblasts from Ossabaw minipigs and Yorkshire pigs were transfected, subcloned, and screened via Sanger sequencing to identify clones carrying the desired allele. Positive clones were pooled and used as donor cells for somatic cell nuclear transfer (SCNT), resulting in the birth of 10 founder piglets from five pregnancies. The PAH modifications did not significantly affect litter size, which remained consistent with historical averages for cloned piglets. The PKU piglets exhibited low birth weight, undetectable PAH enzymatic activity (consistent with the disease phenotype), and required specialised care, including a low-phenylalanine diet, to prevent developmental delays and early mortality. One Ossabaw piglet displayed hypopigmentation, likely linked to impaired Phe metabolism, which is critical for melanin synthesis. To further validate the model, *PAH* gene restoration was achieved using CRISPR-mediated homologous recombination in transfected fibroblasts [31].

In essence, the use of porcine models can improve the accuracy of preclinical studies [30,32]. This is facilitated by an anatomical and physiological structure analogical to that of humans, the similarity in the progression of pathology, and the ability to assess the systemic effect of the disease. However, there are a number of limitations and challenges in using pigs, including the ethical issues of using large animals, a small sample associated with costs and difficulties in keeping, and the existence of alternative model animals.

### 3.3. Zebrafish Models

Zebrafish (*Danio rerio*), due to their significant physiological and genetic homology to mammals, have emerged as a powerful model for studying metabolic disorders [33]. The remarkable degree of evolutionary conservation in genes and proteins shared with humans endows zebrafish with unique advantages in terms of genetic manipulation, cost-efficiency, and high-throughput screening [34]. These characteristics make them an invaluable complement to traditional rodent models. This is particularly evident in instances where the animals do not develop the necessary phenotype of disease progression, as evidenced by previous studies [35,36]. Their accelerated development facilitates metabolic studies as early as 24 h post-fertilisation, enabling efficient investigation of disease mechanisms and therapies at cellular and systemic levels.

**Figure 3:**
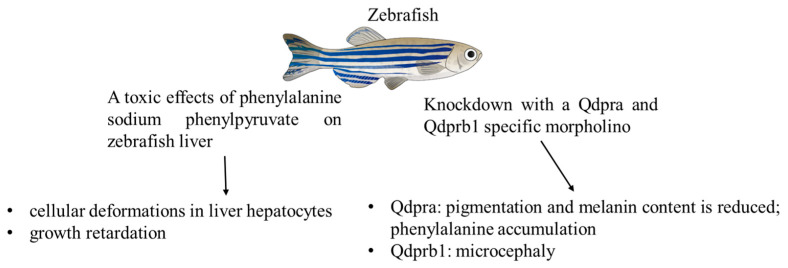
Zebrafish models for the study of phenylketonuria.

Moreover, the use of zebrafish is increasing in the study of the impact of metabolic dysfunction and related therapies [37]. Their fully sequenced genome, ease of genetic manipulation, and high fecundity further enhance their utility as a model for human metabolic syndromes (Figure 3).

A recent study [38] investigated the toxic effects of Phe and its metabolite, sodium phenylpyruvate (SPP), on the zebrafish liver, focusing on developmental and hepatic toxicity. The researchers tested a range of Phe and SPP concentrations on zebrafish embryos to assess their impact. The results obtained revealed that higher doses, particularly concentrations of 10 mg/kg and above, caused significant cellular deformations in liver hepatocytes. In addition to hepatocyte damage, other toxic effects were observed. Zebrafish exposed to elevated levels of SPP demonstrated growth retardation, as indicated by reductions in head length, head width, and caudal fin length. The study emphasises the detrimental effects of high Phe metabolite concentrations on both organ development and overall growth, highlighting the necessity for precise regulation of these compounds in metabolic studies and therapeutic interventions. This study offers significant insights into the impact of Phe metabolites on vertebrate development and liver function.

In a different study [39], the authors established a zebrafish model for dihydropteridine reductase (human: DHPR; mouse/zebrafish: Qdpr), catalysing the recycling of tetrahydrobiopterin (BH4), a cofactor in dopamine, serotonin, and Phe metabolism. The authors used knockdown zebrafish models for their studies: functional knockdown with a Qdpra and Qdprb1-specific morpholino. DHPR deficiency leads to significant neurological and developmental challenges in children, including delayed development, reduced muscle tone (hypotonia), seizures, microcephaly, and elevated Phe levels (hyperphenylalaninemia). Zebrafish have homologs of the *DHPR* gene that are highly conserved with humans (72% homology). A recent study identified two distinct DHPR homologs in zebrafish, Qdpra and Qdprb1, which play different roles. The *Qdpra* gene is associated with the production of BH4 in the liver and melanocytes.

Zebrafish lacking Qdpra exhibit a metabolic phenotype similar to that observed in human patients, including hyperphenylalaninemia. This is evidenced by a reduction in pigmentation and melanin content, as well as an accumulation of Phe, when compared to controls. In contrast, Qdprb1 is essential for neural crest development and cell proliferation, independent of BH4 metabolism. When Qdprb1 is knocked down in zebrafish, they develop microcephaly, resembling the brain atrophy and neurological symptoms observed in patients. This suggests that human DHPR might be involved in both pathways, which could explain the severity of symptoms seen in DHPR deficiency cases.

Zebrafish have emerged as a robust model for studying PKU, offering several distinct advantages over traditional mammalian systems. Their significant genetic similarity to humans allows researchers to observe the direct effects of genetic mutations and metabolic disturbances in real time, owing to their external fertilisation and development. This accessibility, combined with their small size, rapid life cycle, and high fecundity, makes zebrafish ideal for high-throughput screening of therapeutic compounds and genetic modifications. Additionally, their maintenance and genetic manipulation are more cost-effective than rodent models, further supporting their utility in metabolic research.

However, besides the previously listed pros, zebrafish have several cons. One major challenge stems from evolutionary differences between zebrafish and mammals, particularly in metabolic pathways and organ function. Hepatic enzyme activity in zebrafish differs from that of humans, affecting the metabolism of phenylalanine and its derivatives, which may lead to discrepancies in disease modelling. Furthermore, the zebrafish brain, though useful for studying neurotransmitter synthesis, lacks the complexity of mammalian brains, potentially limiting its utility for investigating neurocognitive deficits associated with PKU [40].

Another limitation is related to genetic redundancy in zebrafish due to whole-genome duplication [41,42], which can complicate gene knockdown experiments. Some genes involved in PKU pathogenesis may have paralogs in zebrafish that compensate for mutations, reducing the severity of phenotypes and making it difficult to replicate human disease manifestations. Additionally, zebrafish embryos and larvae are primarily used for high-throughput drug screening, but their application to studying late-onset disease symptoms remains limited. Many human metabolic disorders, including PKU, exhibit progressive pathology that is difficult to capture in fast-developing zebrafish models. These limitations highlight the need to complement zebrafish research with mammalian models for a more comprehensive understanding of PKU.

### 3.4. Avian Models

In addition to traditional models, an avian model (white leghorn chicken) has been created for the elucidation of the molecular mechanisms of congenital heart defects in maternal phenylketonuria (MPKU) conducted at early developmental timepoints [43]. The avian system offers distinct advantages, such as accessibility during embryonic development and the ability to manipulate the embryonic environment, making it a valuable model for investigating the molecular mechanisms underlying MPKU-induced congenital anomalies.

**Figure 4:**
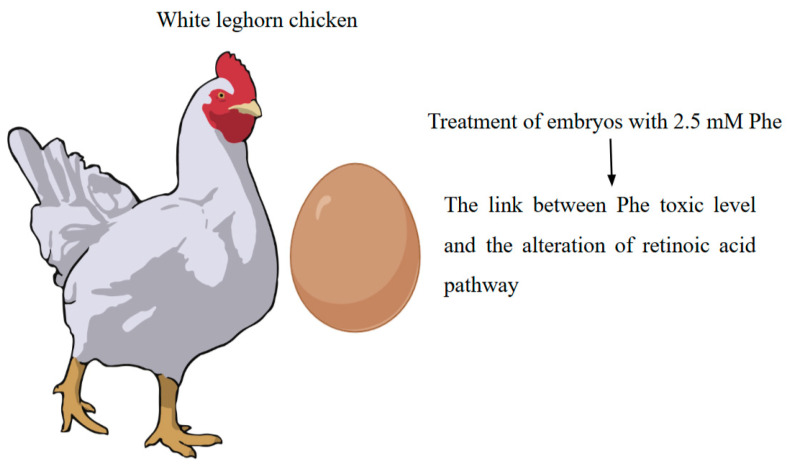
Avian models for the study of phenylketonuria.

A notable study utilised an avian model to explore the impact of elevated maternal Phe levels on early embryonic development. Researchers employed RNA sequencing to analyse differential gene expression at developmental Hamburger and Hamilton (HH) stages: HH10, HH12, and HH14 in chick embryos exposed to high Phe concentrations. These eggs were incubated until HH6, then the eggs were removed from the incubator and, using a needle, were treated with 2.5 mM Phe or vehicle control (PBS) through the blunt end of the egg (Figure 4). After yolk injection, embryos were further incubated until HH 10, 12, or 14. These stages were selected due to the critical changes occurring in the heart at these early development stages [37].

The study identified 633 significantly differentially expressed genes across these stages, with functional annotations revealing associations with clinical phenotypes observed in MPKU, including cardiovascular malformations, craniofacial anomalies, central nervous system defects, and growth retardation. Notably, there was an overrepresentation of genes involved in cardiac muscle contraction; adrenergic signalling in cardiomyocytes; and retinoic acid metabolism, which is necessary for growth and development. These findings suggest a link between elevated Phe exposure and alterations in the retinoic acid pathway, providing insights into the molecular mechanisms of MPKU-induced congenital heart defects [43].

The use of chickens (*Gallus gallus domesticus*) as an avian model in biomedical research has been extensive, primarily due to their accessibility, high fecundity, and well-characterised developmental stages [44]. However, there are notable limitations that researchers must consider when employing chickens in experimental studies. One major issue is the extensive artificial selection that has shaped modern chicken breeds, resulting in physiological and morphological traits that may not be representative of wild-type birds or general vertebrate biology. For example, broiler chickens have been selectively bred for rapid growth and high muscle mass, leading to metabolic disorders such as obesity, hypertension, and skeletal abnormalities, which can confound experimental findings when studying metabolic or developmental diseases.

Additionally, the genetic and phenotypic diversity among chicken breeds complicates the standardisation of experimental results. Different breeds exhibit significant variation in traits such as stress responses, immune function, and metabolic rates, making it challenging to generalise findings across avian species or extrapolate them to mammals. The absence of a “wild-type” domestic chicken further complicates this issue, as comparisons with ancestral species, such as the red jungle fowl, reveal notable differences in physiology and behaviour [45].

Another limitation is the significant difference between avian and mammalian metabolism, particularly in amino acid processing and hepatic enzyme activity. Unlike mammals, birds do not have a urea cycle and instead rely on uric acid as their primary nitrogen excretion pathway, which alters their handling of amino acid imbalances, including Phe accumulation, despite the presence of PAH to convert Phe into tyrosine in the liver and kidneys. This fundamental metabolic difference raises concerns regarding the translatability of PKU research conducted in chickens to human clinical applications [46]. Additionally, the absence of naturally occurring PKU mutations in chickens necessitates the artificial induction of hyperphenylalaninemia, which may not fully replicate the complexity of the human disorder. Therefore, while chickens provide valuable insights, they should be used with caution and ideally in conjunction with mammalian models to enhance the reliability and applicability of research findings.

## 4. Discussion

A number of animal models that have been instrumental in advancing our understanding of phenylketonuria have been described above. These models have facilitated the investigation of disease mechanisms and the development of therapeutic strategies. Each model, including mice and zebrafish, offers unique advantages in mimicking different aspects of the disease. Mouse models, particularly the PAH^enu2^ mouse, provide insights into the genetic and biochemical mechanisms of PKU due to their close physiological and metabolic similarity to humans. Zebrafish models, on the other hand, offer high-throughput screening capabilities and genetic tractability, making them valuable for drug discovery and early-stage metabolic studies.

However, despite their advantages, these models have inherent limitations (Table 2). Rodents, not only mice, but also rats, although widely used, do not perfectly recapitulate all aspects of human PKU, particularly in terms of neurological manifestations. Zebrafish, although highly efficient for genetic studies, differ significantly from mammals in liver metabolism and neural complexity, limiting their translational potential. Avian models, although useful for embryonic research, face challenges due to physiological and metabolic differences that affect their relevance to human PKU. Given these limitations, there is a growing emphasis on integrating alternative research methods, such as patient-derived organoids and computational models, to complement traditional animal studies.

While animal models have been instrumental to PKU research, enabling detailed mechanistic studies and therapeutic development, the field must now evolve to address the limitations of current models and align with emerging ethical and scientific priorities. As precision medicine and gene editing technologies continue to advance, there is an increasing demand for more predictive, human-relevant models.

Animal models remain vital for early-phase studies, particularly in evaluating gene therapy vectors, pharmacokinetics, and systemic toxicity. However, to improve translational relevance, future animal models should incorporate humanised gene constructs [25] and model progressive cognitive decline [47] seen in adult patients. Combining genetic models with non-invasive monitoring tools (e.g., metabolomics) can also help better track disease progression and therapy efficacy over time.

There is growing potential to reduce reliance on animal models by expanding the use of patient-derived induced pluripotent stem cells and 3D brain organoids [48,49]. These systems offer ethical, human-relevant platforms for studying PKU-related neurotoxicity, transporter activity across the blood–brain barrier, and phenylalanine’s impact on neural circuits. For example, iPSC-derived neurons from PKU patients could help validate findings from animal models in a human cellular context.

In order to maintain its relevance and impact, PKU research must adopt a multi-tiered strategy. This strategy should include the refinement of current animal models to enhance their fidelity, the expansion of the use of patient-specific in vitro systems, and the integration of clinical data to guide experimentation driven by hypotheses. By integrating preclinical and clinical research through ethically responsible innovation, the field can more effectively translate laboratory findings into durable therapies for individuals with PKU.

## 5. Conclusions

The landscape of PKU research is evolving, with novel therapeutic strategies and alternative models offering hope for more effective management of the disorder. Continued interdisciplinary research and clinical trials are essential to translate these advances into tangible benefits for people affected by PKU.

It is imperative to comprehend the significance of experimental animal models in PKU research in order to facilitate advancement in both our understanding of the disease and the development of effective therapeutic interventions. This review provides a comprehensive analysis of the current state of PKU animal models, offering a critical evaluation of their respective pros and cons. By systematically examining conventional rodent models alongside alternative species, this work helps bridge existing gaps in determining the most appropriate models for different research applications. This study is a valuable resource for researchers aiming to improve PKU models and develop more effective and ethically responsible treatment strategies.

## Figures and Tables

**Table 1:** Experimental mice model for phenylketonuria.

Animal Model	Creation Method	Main Features	Application	References
BTBR-Pah^enu2^/J (PAH^enu2^)	Chemically Induced Mutation (ENU)	Severe hyperphenylalanemia with serum phenylalanine levels elevated 10–20-fold and urinary ketone concentrations significantly increased in adults. Hypopigmented unless maintained on a low phenylalanine diet.	Behaviour, neurological, growth, metabolism, nervous system, pigmentation, urinary system, reproductive system phenotype.	[22]
B6.BTBR-Pah^enu2^/MalnJ (B6 Pah^enu2^)	Chemically Induced Mutation (ENU)	Hyperphenylalaninemia and other hallmarks of phenylketonuria (PKU) when not maintained on a phenylalanine-free diet. Hyperphenylalaninemia leads to progressive neurological symptoms and seizures. Mice are hypopigmented on a standard diet.	Behaviour/neurological, growth, metabolism, nervous system, pigmentation, urinary system, reproductive system phenotype.	[22]
BTBR-Pah^enu1^/J	Chemically Induced Mutation (ENU)	Consistent with the milder phenotype, homozygotes do not display the pronounced hypopigmentation, or learning and memory deficits. Females do not display a severe maternal effect of smaller and fewer litters and failed survival of pups that are homozygous. Homozygotes have near normal serum phenylalanine levels and elevated brain phenylalanine levels less than twice normal levels.	Homeostasis, metabolism, reproductive system phenotype.	[23]
B6(Cg)-Pah^tm1.1(PAH*R408W)Xiwan^/J (PAH R408W)	Targeted mutation 1.1, Xiao Wan Homologous recombination in C57BL/6 mouse embryonic stem cells was used to replace exon 12 with the orthologous human *PAH* exon 12 sequence including 500 bp of flanking genomic sequence both 5′ and 3′ of exon 12.	Homozygotes have elevated blood phenylalanine levels, exhibit hypopigmentation due to reduced melanin synthesis, and are smaller in size. Blood phenylalanine levels range from approximately 1000 to 1500 μmol/L.	Testing humanised therapeutics.	[24]
C57BL/6J-Pah^em1Xiwan^/J (PAH P281L)	Endonuclease-mediated mutation. CRISPR/cas9 endonuclease-mediated homology-directed repair was used to replace a portion of exon 7.	Homozygotes have elevated blood phenylalanine levels, exhibit hypopigmentation due to reduced melanin synthesis, and are smaller in size. Blood phenylalanine levels range from 1455 to 2242 μmol/L.	Testing humanised therapeutics.	[25]
C57BL/6Smoc-Pah^em1Smoc^ (PAH-KO)	CRISPR/Cas9: The exons 1–13 of mouse *PAH* gene that encode the full-length protein were knocked out in B-Pah KO mice.	The PAH-KO mice showed lower body weights, Summary of blood Phe at various timepoints is shown (*n* = 20/group), summary of blood Tyr at various timepoints is shown (*n* = 20/group).	Behaviour/neurological, growth, metabolism, nervous system, pigmentation phenotype.	[26]
C57BL/6Smoc-Pah^em(R261Q)Smoc^ (Pah-R261Q)	R261Q-PAH mutation was generated by CRISPR/Cas9 genome editing technology	The present study detected a mild form of hyperphenylalaninemia in mice with this mutation. Phenylalanine levels have been found to be elevated to a level twice that which is considered normal. It is noteworthy that the patient does not exhibit other phenotypic abnormalities that are characteristic of classical phenylketonuria, such as hypopigmentation. Males exhibited higher body weight.	Model for studying the tissue-specific effects of phenylalanine metabolism disorders. The model is capable of reproducing a key feature of BH4-responsive forms of HFAs. In addition, it facilitates the study of mechanisms of variability in response to therapy and can be used for preclinical evaluation of new therapies.	[27]

**Table 2:** Pros and cons of experimental models.

Animal Model	Pros	Cons
Mouse models	small size	evolutionary differences between mice and humans
	high reproductive rate	disparities in size
	ease of genetic manipulation	metabolic rate
		lifespan
		immune system
Pig models	anatomy and physiology similar to humans	ethical issues of using large animals
	high sequence and chromosome structure homology with humans	costs and difficulties in keeping
	similar disease progression	small sample size
Zebrafish models	genetic and developmental insights	species differences
	high-throughput screening	liver metabolism differences
	cost-effective	behavioural studies limitations
	disease modelling	
Avian models	accessibility	metabolism differences
	high fecundity	species differences
	well-characterised developmental stages	extensive artificial selection
		genetic and phenotypic diversity

## Abbreviations

The following abbreviations are used in this manuscript:

PAH	Phenylalanine hydroxylase
PKU	Phenylketonuria
Phe	Phenylalanine
Tyr	Tyrosine
NBS	Newborn bloodspot screening
BH4	Tetrahydrobiopterin
HPA	Hyperphenylalaninemia
ENU	N-ethyl-N-nitrosourea
